# Retirement transition and smoking and drinking behaviors in older Chinese adults: Analysis from the CHARLS study

**DOI:** 10.1016/j.pmedr.2023.102408

**Published:** 2023-09-09

**Authors:** Mengyun Luo, Adrian Bauman, Philayrath Phongsavan, Ding Ding

**Affiliations:** a Prevention Research Collaboration, Sydney School of Public Health, Faculty of Medicine and Health, The University of Sydney, Sydney, NSW 2006, Australia; b Charles Perkins Centre, The University of Sydney, Sydney, NSW 2006, Australia; c School of Public Health, School of Medicine, Shanghai Jiao Tong University, Shanghai 200025, People’s Republic of China

**Keywords:** Retirement transition, Low- to middle-income country, Tobacco smoking, Excessive drinking, Longitudinal research

## Abstract

**Introduction:**

Smoking and drinking are important public problems and a substantial part of work culture in mainland China. However, little is known about the effect of retirement on these behaviors. Thus, this study examined the relationships between retirement transition, smoking and excessive drinking among older Chinese adults.

**Methods:**

Repeated longitudinal data from a nationally representative sample of Chinese adults were collected in 2011, 2013, 2015 and 2018. Respondents completed a structured questionnaire regarding work status and health behaviors. Modified mixed-effects Poisson regression models were used to explore the associations, with additional analyses stratified by gender.

**Results:**

Of the 10,378 participants included, 62.6% and 20.1% of men reported current smoking and excessive drinking at study entry; compared to 4.4% and 1.5% of women, respectively. There was no significant association between retirement and smoking. We found a dose–response relationship between time in retirement and excessive drinking in the adjusted model where those who retired >= 2 years ago had a 30% lower risk of excessive drinking (risk ratio (RR) = 0.70, 95% confidence interval (CI) = 0.56–0.86), and those who retired < 2 years ago had a 16% lower risk of excessive drinking (RR = 0.84, 95%CI = 0.73–0.97), compared with those who remained working. This pattern remained when analyzed separately for men and women, although not all results reached statistical significance.

**Discussion:**

Chinese older adults are more likely to reduce drinking following retirement. Such evidence supports the positive framing of retirement in public discourse and the need for workplace interventions to address excessive drinking in China.

## 1 Introduction

Smoking and excessive drinking are major health issues in China, ranking the 1st and 8th contributing risk factors to disability-adjusted life years lost. (GBD, 2019) In China, both risk behaviors are much more common in men than in women, with 50.5% of men and 2.1% of women estimated to be current smokers, (Chinese Center for Disease Control and Prevention, 2020) and 56% of men and 15% of women current drinkers. Among those who drink, 57% of the male drinkers and 27% of the female drinkers reported excessive drinking. (Li et al., 2011).

Retirement transition is a universal experience and is widely considered as a major life transition. Previous research suggests that retirement may have profound effects on lifestyle and health due to changes in time availability, daily routines, work-related stress, identity/purpose, financial resources and social interactions. (Ding et al., 2016) Findings from previous studies suggest that the effects of retirement on health differed by the specific outcomes examined and the study population. The existing evidence on the effects of retirement on smoking and drinking behaviors is mixed, and is mostly based on Western data. For example, Kim, Motegi, and Ding found that retirees were less likely to be heavy smokers or drinkers compared to their employed counterparts among the older population in Korea, Japan, and Australia while others found either a positive association between retirement and smoking/alcohol intake or no significant association at all. (Ding et al., 2016, Kim et al., 2016, Motegi et al., 2016, Halonen et al., 2017, Xue et al., 2020).

To date, evidence on retirement and smoking/drinking behaviors in China, a rapidly aging country with an early mandatory retirement age (∼60 for men, ∼55 for women), is still limited. In China, both smoking and drinking behaviors are deeply rooted in the social environment. They are considered as such important social and cultural practices that offering cigarettes and alcoholic drinks are used to break down social barriers with strangers, foster friendships with co-workers and build bonding with business partners. (Wu et al., 2022) Meanwhile, the tobacco and alcohol industries take advantage of such cultural norms by promoting their products as ‘facilitators’ or ‘proof’ for friendships, social bonds and business partnerships. (Hu et al., 2022) A recent study found that around 97% of current smokers reported sharing and 90% reported gifting cigarettes in China. (Liao et al., 2022) Due to the social and cultural context of smoking and drinking, one may hypothesize that retirement may result in changes in both behaviors due to different social prompts, pressure, and reinforcement for smoking and drinking. Evidence from China, an aging country with an early mandatory retirement age (∼60 for men, ∼55 for women), different social environments and gender-specific lifestyle risk profiles, could offer unique insights. This study aims to examine the relationship of retirement transitions with smoking/ excessive drinking in older Chinese men and women.

## 2 Methods

### 2.1 Participants and settings

The China Health and Retirement Longitudinal Study (CHARLS) is a nationally representative longitudinal survey of Chinese adults aged 45 and up. (Zhao et al., 2014) The CHARLS 2011 baseline survey was conducted in 28 provinces across the country, using a multistage, stratified, probability proportionate-to-size sampling strategy. Repeated data were then collected in 2013, 2015 and 2018. Ethical approval was obtained from the Biomedical Ethics Review Committee of Peking University, and all participants provided written informed consent.

Data analysis of the current study was restricted to those who provided complete data for employment status in at least two waves and who reported ‘working’ upon entering the study (in 2011, 2013 or 2015), so that retirement transition status could be ascertained. The final study sample includes 33,570 observations from 10,378 participants.

### 2.2 Measurement

Based on reported working status and retirement time, we classified employment status into “working”, “retired for < 2 years”, “retired for 2–4 years” and “retired for 4–7 years” (7 years was the maximum time since retirement between 2011 and 2018). Due to the small sample size of participants who retired for 4–7 years, we decided to combine the last two groups together to be “retired for >= 2 years”.

Current smoking was defined as having ever smoked 100+ cigarettes (lifetime), and currently smoking. Participants reported the frequency of consuming each type of alcohol (beer, wine/rice wine, liquor) in a month and the volume consumed on one occasion. The total amount of pure alcohol consumed (in grams) was estimated using the formula “volume(ml) × concentration(%) × 0.8”, based on the following alcohol content by volume: beer 4%, wine/rice wine 13.5%, liquor 45.5%. (Ge et al., 2018) “Excessive drinking” was defined as consuming an average of 25 + grams/day for men and 15 + grams for women, according to the Chinese Dietary Guidelines. (Chinese Nutrition Society, 2016).

Covariates considered included gender (men/women), age (continuous in years), marital status (married/not married), educational attainment (no formal education/primary school or below/middle school or above), and place of residence (rural/urban). Binary variables (yes/no) were created for diagnosed chronic diseases, where participants responded to a pre-defined list of conditions including hypertension, diabetes or high blood sugar, cancer (excluding non-malignant skin cancers), chronic lung disease, heart disease, stomach or other digestive diseases, and arthritis or rheumatism.

### 2.3 Statistical analysis

Descriptive statistics were presented as mean values and standard error for continuous variables, and numbers and weighted percentages for categorical variables. Since each individual had multiple measurements at different time points and individuals were clustered within communities, we examined the associations between retirement and smoking/excessive drinking using modified mixed-effects Poisson regression models, (Chen et al., 2018) with random intercepts across individuals and fixed effects of retirement on smoking/excessive drinking. We constructed the models sequentially, with Model 1 unadjusted, Model 2 adjusted for socio-demographic characteristics and Model 3 additionally adjusted for chronic disease variables. Post-stratification weights were applied to ensure population-representativeness. Considering the well-documented differences between men and women in smoking and drinking behaviors in the Chinese context, (Chinese Center for Disease Control and Prevention, 2020, Li et al., 2011) we tested a gender × retirement multiplicative interaction term followed by gender-stratified analysis. All analyses were conducted using R version 4.2.1 and Stata/BE 17.0 for Windows.

## 3 Results

Table 1 presents descriptive statistics of the final sample (n = 10,378). Included participants were on average 54.5 years old with a relatively balanced gender distribution, and over 90% of the participants were married upon entry. Around 90% of men and 63% of women received formal school education. Most participants lived in rural areas. Of the chronic diseases examined, arthritis, digestive problems and hypertension were the most common. The prevalence of smoking and excessive drinking was 62.6% and 20.1% in men and 4.4% and 1.5% in women, respectively.

**Table 1:** Descriptive statistics of 10,378 Chinese older population from the CHARLS study at baseline between 2011 and 2015 (mean values and standard error; weighted proportion).

Characteristics	Total (n = 10,378)	Men (n = 5,027)	Women (n = 5,351)
Men; n(%)	5,027 (48.8)		
Age; mean (SE)	54.51 (0.11)	55.10 (0.16)	53.95 (0.15)
Married; n(%)	9,578 (91.9)	4,674 (92.7)	4,904 (91.2)
Educational attainment; n(%)			
No formal education	2,637 (24.1)	575 (10.8)	2,062 (36.7)
Primary school or below	4,330 (40.6)	2,245 (43.3)	2,085 (37.9)
Middle school or above	3,411 (35.4)	2,207 (45.9)	1,204 (25.4)
Rural residence; n(%)	7,380 (64.6)	3,547 (63.9)	3,833 (65.2)
Have been diagnosed with the following disease by a doctor:			
Hypertension; n(%)	1,898 (17.7)	888 (17.8)	1,010 (17.7)
Diabetes or high blood sugar; n(%)	382 (3.6)	170 (3.4)	212 (3.7)
Cancer (excluding minor skin cancers); n(%)	77 (0.7)	16 (0.3)	61 (1.1)
Chronic lung disease; n(%)	819 (7.2)	455 (8.3)	364 (6.2)
Heart disease; n(%)	807 (7.3)	325 (6.2)	482 (8.3)
Stomach or other digestive disease; n(%)	2,393 (21.9)	1,049 (20.2)	1,344 (23.6)
Arthritis or rheumatism; n(%)	3,400 (30.7)	1,470 (27.9)	1,930 (33.3)
Smoking; n(%)	3,495 (32.8)	3,238 (62.6)	257 (4.4)
Excessive drinking; n(%)	1,162 (10.6)	1,077 (20.1)	85 (1.5)
Note: CHARLS, China Health and Retirement Longitudinal Study; SE, standard error.			

Table 2 presents the associations of retirement with smoking and excessive drinking. There was a significant inverse relationship between time since retirement and smoking behavior in the unadjusted model. However, that association disappeared after adjusting for socio-economic status and chronic diseases. A dose–response relationship was observed between retirement and excessive drinking in all models, despite minimal attenuation in magnitude after adjustment in Models 2 and 3. In Model 3, compared with those who remained working, those who retired < 2 years ago and more than 2 years ago had 16% and 30% reductions in the risk of excessive drinking, respectively. Gender × retirement interaction was significant for smoking (p = 0.021 for retired for < 2 years vs working, p = 0.225 for retired for >= 2 years vs working), while not significant for excessive drinking (pooled p = 0.377). The dose–response pattern remained similar when analyzing men and women separately in the stratified analysis, though not reaching statistical significance for people who retired < 2 years.

**Table 2:** Association of retirement with smoking and excessive drinking among 10,378 Chinese older population from the CHARLS study between 2011 and 2018.

	Total (n = 10,378)			Men (n = 5,027)			Women (n = 5,351)		
	Model 1	Model 2	Model 3	Model 1	Model 2	Model 3	Model 1	Model 2	Model 3
	RR(95 %CI)	RR(95 %CI)	RR(95 %CI)	RR(95 %CI)	RR(95 %CI)	RR(95 %CI)	RR(95 %CI)	RR(95 %CI)	RR(95 %CI)
Smoking									
Working	1	1	1	1	1	1	1	1	1
Retired for < 2 years	0.95 (0.91–0.99)	1.03 (0.98–1.09)	1.04 (0.98–1.09)	0.99 (0.94–1.05)	1.01 (0.96–1.07)	1.02 (0.97–1.08)	1.16 (1.02–1.31)	1.09 (0.96–1.24)	1.10 (0.97–1.25)
Retired for >= 2 years	0.87 (0.81–0.93)	1.02 (0.94–1.11)	1.04 (0.95–1.13)	1.00 (0.92–1.09)	1.02 (0.94–1.11)	1.05 (0.96–1.14)	1.14 (0.96–1.35)	0.97 (0.80–1.17)	0.95 (0.78–1.15)
Excessive drinking									
Working	1	1	1	1	1	1	1	1	1
Retired for < 2 years	0.79 (0.68–0.91)	0.83 (0.72–0.96)	0.84 (0.73–0.97)	0.86 (0.74–0.99)	0.87 (0.75–1.01)	0.88 (0.76–1.02)	0.68 (0.42–1.13)	0.62 (0.37–1.04)	0.62 (0.37–1.03)
Retired for >= 2 years	0.59 (0.48–0.73)	0.67 (0.54–0.83)	0.70 (0.56–0.86)	0.72 (0.58–0.91)	0.75 (0.59–0.94)	0.78 (0.62–0.98)	0.59 (0.32–1.10)	0.42 (0.22–0.79)	0.43 (0.23–0.80)
Model 1: unadjusted. Model 2: adjusted for socio-demographic variables only, including gender (in overall analysis), age, marital status, educational attainment, and place of residence. Model 3: adjusted for both socio-demographic and chronic disease variables, including gender (in overall analysis), age, marital status, educational attainment, place of residence, hypertension, diabetes or high blood sugar, cancer (excluding minor skin cancers), chronic lung disease, heart disease, stomach disease or other digestive disease, and arthritis or rheumatism. Note: All the results were weighted. CHARLS, China Health and Retirement Longitudinal Study; CI, confidence interval; RR, risk ratio.									

## 4 Discussion

Our study found an inverse association between time since retirement and excessive drinking, but not smoking behavior in older Chinese adults. This finding confirmed the protective effect of retirement on some lifestyle behaviors even after adjusting for a range of confounders. (Ding et al., 2016, Smeaton et al., 2017).

Previous research suggested that retirement could offer a unique opportunity for positive lifestyle change, (Smeaton et al., 2017) potentially explained by changes in social networks, roles, and stressors. (Bamberger, 2015) Several factors may explain our findings. First, in China, drinking behavior in a workplace setting with colleagues or clients is considered necessary for building business connections (‘Guanxi关系’), securing business deals and career advancement. (Hu et al., 2022) Thus, from a social network’s perspective, retirement reduces exposure to socially reinforcing environments for drinking, especially among men. (Knox et al., 2019) Second, self-identification with one’s role could affect their post-retirement adjustment. (Wang et al., 2011) In China, around 58% of older adults aged 45–79 years care for their grandchildren. (Ko and Hank, 2014) Therefore, the idea of being a good role model may motivate grandparents to cease excessive drinking. (Zhou et al., 2017) Third, removal of work-related stress may lead to reduction in stress-coping behaviors, such as drinking. Moreover, reduced income could also limit the financial resources to buy alcoholic drinks. (Bareham et al., 2019) Finally, retirement has been considered as a potential window of opportunity for health behavior change, prompting older adults to rethink their lifestyle. In China, ‘Yangsheng养生’ (meaning ‘health-keeping practices’) is a popular concept among retirees and older adults, which may prompt them to quit unhealthy behaviors. (Smeaton et al., 2017, Sun, 2016).

Though all the factors mentioned above could also be applied to smoking, the main difference between tobacco and alcohol consumption in China is that smoking is ubiquitous and less setting-specific. People who are addicted to nicotine may be able to smoke nearly anytime and anywhere without social prompts or specific occasions. As the largest producer and consumer of tobacco in the world, over half of the Chinese men are current smokers and cigarettes are served as “social currency”, to the degree that cigarettes are exchanged on a daily basis as gifts. (Ding and Hovell, 2012) Therefore, the sole removal from particular social settings may not be sufficient for reducing smoking in China. Instead, comprehensive tobacco control policies, such as taxation, plain packaging, and smoking bans in public places, are needed to fundamentally address the high smoking prevalence among Chinese men.

## 5 Limitations

Several limitations need to be acknowledged. First, retirement status was self-reported and information regarding participants’ jobs (e.g., blue- vs white-collar jobs, occupation, work demands), which could be potential effect modifiers, was not collected. Thus, endogeneity issues cannot be addressed in the current study. Second, measures of some potential confounders, such as social networks, were not available. Third, due to the limited follow-up period, only short-to-medium-term (up to seven years) effects of retirement could be tested. Third, our analyses were underpowered to test the full dose–response relationships between time since retirement and smoking/drinking outcomes due to the small sample sizes in the “retired for 4–7 years” group. Fourth, Future research with a larger sample size and long follow-up periods is needed. Finally, the findings may not be generalizable to populations outside of mainland China.

## 6 Conclusion

We found that older Chinese adults were likely to reduce drinking, but not quit smoking following retirement. Such evidence supports a positive framing around retirement in public discourse and highlights the need for evidence-based interventions to address the workplace risk of drinking in China.

## CRediT authorship contribution statement

**Mengyun Luo:** Conceptualization, Methodology, Formal analysis, Writing – original draft. **Adrian Bauman:** Writing – review & editing, Supervision. **Philayrath Phongsavan:** Writing – review & editing, Supervision. **Ding Ding:** Conceptualization, Writing – review & editing, Supervision.

## Declaration of Competing Interest

The authors declare that they have no known competing financial interests or personal relationships that could have appeared to influence the work reported in this paper.

## Data Availability

Data will be made available on request.

## References

[b0005] Bamberger P.A. (2015). Winding Down and Boozing Up: The Complex Link Between Retirement and Alcohol Misuse. Work Aging Retirem..

[b0010] Bareham B.K., Kaner E., Spencer L.P., Hanratty B. (2019). Drinking in later life: a systematic review and thematic synthesis of qualitative studies exploring older people's perceptions and experiences. Age Ageing.

[b0015] Chen W., Qian L., Shi J., Franklin M. (2018). Comparing performance between log-binomial and robust Poisson regression models for estimating risk ratios under model misspecification. BMC Med. Res. Method..

[b0020] Chinese Center for Disease Control and Prevention (2020). 2018 China Adult Tobacco Survey Report. https://extranet.who.int/ncdsmicrodata/index.php/catalog/803/download/5569.

[b0025] Chinese Nutrition Society. The Chinese Dietary Guidelines 2016. http://dg.cnsoc.org/article/04/8a2389fd5520b4f30155be01beb82724.html. Accessed December 5, 2021.

[b0030] Ding D., Hovell M.F. (2012). Cigarettes, social reinforcement, and culture: a commentary on “Tobacco as a social currency: cigarette gifting and sharing in China”. Nicotine Tob. Res..

[b0035] Ding D., Grunseit A.C., Chau J.Y., Vo K., Byles J., Bauman A.E. (2016). Retirement-A Transition to a Healthier Lifestyle?: Evidence From a Large Australian Study. Am. J. Prev. Med..

[b0040] GBD 2019 Risk Factors Collaborators, 2020. Global burden of 87 risk factors in 204 countries and territories, 1990-2019: a systematic analysis for the Global Burden of Disease Study 2019. Lancet. 396, 1223-1249. https://doi.org/10.1016/S0140-6736(20)30752-2. 10.1016/S0140-6736(20)30752-2 PMC7566194 33069327

[b0045] Ge S., Wei Z., Liu T., Wang J., Li H., Feng J., Li C. (2018). Alcohol Use and Cognitive Functioning Among Middle-Aged and Older Adults in China: Findings of the China Health and Retirement Longitudinal Study Baseline Survey. AlcoholClin Exp Res..

[b0050] Halonen J.I., Stenholm S., Pulakka A., Kawachi I., Aalto V., Pentti J., Lallukka T., Virtanen M., Vahtera J., Kivimäki M. (2017). Trajectories of risky drinking around the time of statutory retirement: a longitudinal latent class analysis. Addiction.

[b0055] Hu A., Jiang H., Dowling R., Guo L., Zhao X., Hao W., Xiang X. (2022). The transition of alcohol control in China 1990–2019: Impacts and recommendations. Int. J. Drug Policy.

[b0060] Kim J., Cha S.-E., Kawachi I., Lee S. (2016). Does Retirement Promote Healthy Behaviors in Young Elderly Korean People?. J. Behav. Health.

[b0065] Knox J., Schneider J., Greene E., Nicholson J., Hasin D., Sandfort T., Huerta-Quintanilla R. (2019). Using social network analysis to examine alcohol use among adults: A systematic review. PLoS One.

[b0070] Ko P.C., Hank K. (2014). Grandparents caring for grandchildren in China and Korea: findings from CHARLS and KLoSA. J. Gerontol. B Psychol. Sci. Soc. Sci..

[b0075] Li Y., Jiang Y., Zhang M., Yin P., Wu F., Zhao W. (2011). Drinking behaviour among men and women in China: the 2007 China Chronic Disease and Risk Factor Surveillance. Addiction.

[b0080] Liao Y., Tang J., McNeill A., Kelly B.C., Cohen J.E. (2022). Impact of cigarette package warnings on attitudes towards sharing and gifting cigarettes in China: a nationwide study of smokers and non-smokers. Tob. Control.

[b0085] Motegi, H., Nishimura, Y., Terada, K. (2016). Does Retirement Change Lifestyle Habits? The Japanese Economic Review, 67(2):169-191. http://doi.org/ 10.1111/jere.12104.

[b0090] Smeaton D., Barnes H., Vegeris S. (2017). Does Retirement Offer a “Window of Opportunity” for Lifestyle Change? Views From English Workers on the Cusp of Retirement. J. Aging Health.

[b0095] Sun W. (2016). Regimes of healthy living: The reality of ageing in urban China and the cultivation of new normative subjects. J. Consum. Cult..

[b0100] Wang M., Henkens K., van Solinge H. (2011). A review of theoretical and empirical advancements. Am. Psychol..

[b0105] Wu D., Jiao G., Hu H., Zhang L., Huang L., Jiang S. (2022). Cigarette sharing and gifting in China: Patterns, associated factors, and behavioral outcomes. Tob. Induc. Dis..

[b0110] Xue B., Head J., McMunn A., Heyn P.C. (2020). The Impact of Retirement on Cardiovascular Disease and Its Risk Factors: A Systematic Review of Longitudinal Studies. Gerontologist.

[b0115] Zhao Y., Hu Y., Smith J.P., Strauss J., Yang G. (2014). Cohort profile: the China Health and Retirement Longitudinal Study (CHARLS). Int. J. Epidemiol..

[b0120] Zhou J., Mao W., Lee Y., Chi I. (2017). The Impact of Caring for Grandchildren on Grandparents' Physical Health Outcomes: The Role of Intergenerational Support. Res. Aging.

